# Identification of Nematicidal Metabolites from *Purpureocillium lavendulum*

**DOI:** 10.3390/microorganisms10071343

**Published:** 2022-07-02

**Authors:** Rui Liu, Zheng-Xue Bao, Guo-Hong Li, Chun-Qiang Li, Shao-Lin Wang, Xue-Rong Pan, Ke-Qin Zhang, Pei-Ji Zhao

**Affiliations:** State Key Laboratory for Conservation and Utilization of Bio-Resources in Yunnan, School of Life Sciences, Yunnan University, Kunming 650091, China; ruiliu991@outlook.com (R.L.); bzx357489@126.com (Z.-X.B.); ligh@ynu.edu.cn (G.-H.L.); chunqianglee@outlook.com (C.-Q.L.); wsl774799443@outlook.com (S.-L.W.); xuerong_pan@ynu.edu.cn (X.-R.P.); kqzhang1@ynu.edu.cn (K.-Q.Z.)

**Keywords:** *Purpureocillium lavendulum*, lipoxygenase, hydroxylated fatty acids, nematicidal activity, *Meloidogyne incognita*, transcriptome data

## Abstract

*Purpureocillium lavendulum* is a fungus with promising biocontrol applications. Here, transcriptome data acquired during the infection of *Caenorhabditis elegans* by *Purpureocillium lavendulum* showed that the transcription of metabolite synthesis genes was significantly up-regulated after 24 and 48 h of the fungus-nematode interaction. Then, the up-regulated transcription level of lipoxygenase was confirmed by RT-qPCR. The ultra-performance liquid chromatography-mass spectrometry (UPLC-MS) analysis of differential metabolites revealed that this interaction resulted in the emergence of new metabolites or enhanced the production of metabolites. The results of the UPLC-MS analysis and the nematicidal assay were used to establish optimal culturing conditions under which 12 metabolites, including 3 hydroxylated C_18_ fatty acids and 9 steroids, were isolated and identified. Among them, hydroxylated fatty acids showed pronounced nematicidal activity against *Meloidogyne incognita*, and two degradative sterols showed chemotaxis activity to *M. incognita*. This study lays a foundation for the function of lipoxygenase and its products during the infection of *Purpureocillium lavendulum*.

## 1. Introduction

As agricultural pests of global significance, plant-parasitic nematodes, particularly *Meloidogyne* spp. and *Heterodera* spp., cause large crop losses annually [1]. Nematophagous fungi are a diverse group of fungal species that can capture or infect nematodes. These fungi represent an important source of potential biocontrol agents against plant-parasitic nematodes [2,3]. *Purpureocillium lavendulum* [4] is a fungal species discovered recently and is a close relative of the nematophagous fungus *P. lilacinum* (formerly known as *Paecilomyces lilacinus*), which has been the most widely used species for controlling nematodes and harmful insects [5,6,7]. One obvious difference between the two species is that *P. lavendulum* cannot grow at above 35 °C and is therefore not considered an infectious threat to humans, while *P. lilacinum* can grow well at above 35 °C [8]. Lacking growth above 35 °C makes *P. lavendulum* safer as a biological control agent.

Research on the mechanism of such biocontrol has mainly focused on the activity of extracellular enzymes [9,10] and metabolites profiling. Tadashi isolated leucinostatins (non-ribosomal peptides) from the fermentation broth of *Purpureocillium* spp. and demonstrated that the members of this novel antibiotics class inhibit the proliferation of certain gram-positive bacteria, fungi, and even tumor cells [11]. Another work showed that leucinostatins can be produced by different strains of *P. lilacinum* and are highly toxic to nematodes [12]. In addition, other compounds including fatty acids [12], phenolic acids, sesquiterpenoids [13], and paecilomide [14] were isolated from *Purpureocillium* species and were shown to exhibit nematicidal activities.

As the effects of most direct biocontrol agents are affected by soil type, climatic conditions, and other factors defining a specific deployment region, the identification of nematicidal components and their action mechanisms is important for improving control efficiency. The biosynthesis of natural products has been studied for more than 100 years, with the related research currently focusing on the localization, cloning, and functional identification of synthetic genes. Although several active compounds have been isolated from *Purpureocillium* spp. (previously reported as *Paecilomyces lilacinus*) and identified [15], their roles in the infestation of nematodes by *P. lavendulum* remain unknown. The possible involvement of small molecular compounds in this infestation process may be probed by subjecting *P. lavendulum* interacting with nematodes to transcriptomic data and metabolite difference analyses. Fungi and bacteria can produce various secondary metabolites, depending on environmental conditions such as temperature and medium composition [16], a phenomenon known as “one strain many compounds” [17]. Herein, transcriptomic data analysis showed that parts of metabolite synthesis genes were significantly up-regulated after 24 and 48 h interactions of *P. lavendulum* and *Caenorhabditis elegans*. Moreover, ultra-performance liquid chromatography-mass spectrometry (UPLC-MS) profiling showed that this interaction resulted in the emergence of new metabolites and changes in the abundances of existing metabolites. Several sterols and three hydroxylated fatty acids were obtained from *P. lavendulum* under the optimal culturing conditions established through transcriptomic data and different compound analyses. The corresponding bioassay using *M. incognita* indicates that hydroxylated fatty acids showed nematicidal activity to *M. incognita*, while sterols induced positive chemotaxis.

## 2. Materials and Methods

### 2.1. Materials

*P. lavendulum* YMF1.00683 was isolated from soil in Yunnan Province, China [18]. *M*. *incognita* was obtained from the roots of tomatoes grown in E’shan County in Yunnan Province. *C. elegans* N2 was maintained on a nematode growth medium with *Escherichia coli* strain OP50.

*M. incognita* egg masses were handpicked from tomato root galls, surface-sterilized in 1% NaClO solution for 4 min, rinsed three times with distilled water (dH_2_O), placed in a Petri dish with water, and incubated in the dark at 25 °C to prepare second-stage juveniles (J2s). The newly hatched J2s were collected daily after hatching. The worm concentration was adjusted to 2 × 10^4^ mL^−1^ according to experimental needs. 

### 2.2. Infection of C. elegans by P. lavendulum

*P*. *lavendulum* grown on PDA plates for seven days at 28 °C was harvested by cutting into pieces, vibrated with 0.1% Tween-80 for 30 min at 100 RPM, and filtered through sterilized four-layer filter paper to obtain a spore suspension. The spores were counted, and their concentration was adjusted to 10^6^ mL^−1^. A 200 μL aliquot of the spore suspension was evenly spread on a cellophane-covered water agar plate, which was then covered and cultured at 28 °C for three days to germinate spores. *C. elegans* at the L1 stage was washed with sterile ddH_2_O, and the concentration was adjusted to ~1500 mL^−1^. Three experimental groups were set up for the differential metabolite analysis of the infection of *C. elegans* by *P. lavendulum*, group P (*P*. *lavendulum* culture; 100 μL of water was added to each cellophane-covered water ager plat), group C (*C*. *elegans* culture; 100 μL of *C. elegans* dispersion was added to each cellophane-covered water agar plate), and group PC (100 μL of *C. elegans* dispersion was added into the germinated spores’ plates). For all groups, samples were collected at 24, 48, 72, 96, and 120 h, soaked in methanol for three days, steam-dried, and dispersed in methanol for UPLC-MS analysis. Samples for the transcriptomic analysis of *P*. *lavendulum* interacting with *C. elegans* were prepared by the same method for each sample with 0.3 g of fresh weight mycelia (or/and worm), and the mycelia were washed with ddH_2_O. Three replicates were conducted for each time point. The transcriptome sequencing was assisted by the BioMarker Company (Beijing, China).

### 2.3. Analysis of Gene Transcription by RT-qPCR

RNA was extracted using the AxyPrep Multisource Total RNA Miniprep Kit (AXYGEN, Union City, CA, USA), and cDNA was then synthesized on ice using TaKaRa’s PrimeScript^TM^ RT Reagent Kit with a gDNA Eraser (Perfect Real Time). β-Tubulin was used as an internal reference gene, group P was used as the control for each interaction time, and group PC was used as the experimental group. ON184318 (encoding a linoleate diol synthase and GenBank: ON184318), ON184319 (encoding chitinase and GenBank: ON184319), and ON184320 (encoding a 3-hydroxy-3-methylglutaryl-coenzyme A reductase and GenBank: ON184320) were used as the target genes. The Roche LightCycler^®^ 480 SYBR Green I Master was used for RT-qPCR detection by the Roche LightCycler 480 real-time fluorescent quantitative PCR instrument. At last, the RT-qPCR primers used are as follows: β-tubulin-F: 5′-CACGCTGCTCATCTCCAAG-3′; β-tubulin-R: 5’-CAG- GTAGTTGAGGTCGCCATA-3′; ON184318-F: 5′-ACGCTCAAGGACATTGTCAAGT-3′; ON184318-R: 5′-CCACAGCTCGCCGATGAA-3′; ON184319-F: 5′-TCTGCGAGTAAT- AGGCATGGAC-3′; ON184319-R: 5′-AACGACGTGCTCCTCCTCAA-3′; ON184320-F: 5′- AGCGTCATCCTGCTGTCCG-3′; ON184320-R: 5′-GATCTCGTAGCCCTTGTCCTTG-3′.

### 2.4. UPLC-MS Analysis

UPLC-MS analysis was performed on a Dionex UltiMate 3000 LC system coupled with a Q-Exactive Orbitrap mass spectrometer (San Jose, CA, USA) (Thermo, Bremen, Germany) with an electrospray ionization (ESI) source. Separation was performed on a Hypersil Gold column (100 mm × 2.1 mm, Thermo Fisher Scientific, Waltham, MA, USA) with a particle size of 1.9 µm at an LC flow rate of 300  μL min^−1^ and a column temperature of 40 °C. Mobile phase A was 0.1% formic acid in water, and mobile phase B was 0.1% formic acid in methanol. The 30 min gradient for the positive ESI mode was set as follows: 0–2 min, 5% solvent B; 2–22 min, 5–95% solvent B; 22–25 min, 95% solvent B; and 25–30 min, 5% solvent B. The injection volume was 10 μL, and each sample was injected in triplicate. For normal detection, the ESI source parameters were set to capillary temperature = 350 °C, sheath gas flow rate = 35 arbitrary units (a.u.), auxiliary gas flow rate = 8 a.u., spray voltage = 3.5 kV, and a full MS resolution of 70,000, whereas fatty acid detection was performed at sheath gas = 25 a.u., auxiliary gas =10 a.u., auxiliary gas heater temperature = 220 °C, spray voltage = −2.8 kV, and capillary temperature = 350 °C. For the full scan, the automatic gain control (AGC) target and maximum injection time (IT) were 3 × 10^6^ and 100 ms, respectively, with a resolution of 70,000. For parallel reaction monitoring (PRM), the resolution was set at 17,500, and the AGC target and maximum IT were 2 × 10^5^ and 100 ms, respectively. The normalized collision energy (CE) was set as 30%. The LC–MS instrument was controlled using Thermo Scientific Xcalibur 4.1 software (San Jose, CA, USA).

### 2.5. Optimization of P. lavendulum Culturing Conditions

Five solid media (CMA (20.0 g corn, 10.0 g glucose, 0.4 g peptone, 1.0 g vitamin B, 15.0 g agar, 1 L water), PDA (200.0 g potato, 20.0 g glucose, 15.0 g agar, 1 L water), YMG (4.0 g yeast extract, 20.0 g glucose, 15.0 g agar, 1 L water), Oat (300.0 g oatmeal, 1 mL trace element solution 1, 15.0 g agar, 1 L water), and TYGA (10.0 g tryptone, 5.0 g yeast extract, 10.0 g glucose, 5.0 g molasses, 15.0 g agar, 1 L water)) were used to optimize culture conditions. Trace element solution 1 (2.2 g ZnSO_4_·7H_2_O, 1.1 g H_3_BO_3_, 0.5 g MnCl_2_·4H_2_O, 0.16 g FeSO_4_·7H_2_O, 0.16 g CoCl_2_·5H_2_O, 0.16 g CuSO_4_·5H_2_O, 0.11 g (NH_4_)_6_Mo_7_O_4_·4H_2_O, 5 g Na_2_EDTA, 100 mL water) was adjusted to a pH of 6.5 with KOH. Solid media (200 mL) were divided into 10 petri dishes. Culturing was performed at 28 °C for 21 days. The solids were extracted exhaustively three times by EtOAc/MeOH/AcOH (80:15:5, *v*/*v*/*v*), and the extracts were examined by LC-MS. The nematicidal activity of some crude extracts was also considered (dead nematodes at 12, 24, and 48 h of 400 ppm crude extracts were counted).

### 2.6. Metabolite Isolation and Purification

Based on the results of the culturing condition optimization, we selected oat agar as the medium for the culturing of P. lavendulum at 28 °C for 21 days, with the total fermentation volume equaling 30 L. After soaking, the soaking solution was filtered out from four layers of gauze and then decompressed and dried at 45 °C to obtain the extract. The extract was dissolved in distilled water (6 L), and the solution was successively extracted with the same volumes of petroleum ether, ethyl acetate, and n-butanol. Each of the three extracts was concentrated, and the mass of the ethyl acetate fraction was determined as 252.4 g.

The EtOAc fraction (252.4 g) was subjected to silica gel column chromatography (100–200 mesh, Qingdao Marine Chemical Factory; 120 × 10 cm column) using a petroleum ether–EtOAc gradient (500:1 to 8:2) and a chloroform–MeOH gradient (100:1 to 0:100) to give ten fractions (Fr.1-Fr.10). Fr.4 (1.044 g) was chromatographed on a silica gel column (100 × 4 cm; petroleum ether-acetone, 200:1, 100:1, 50:1, 20:1, 10:1, 9:1, 8:2) to give nine fractions (Fr.4.1-Fr.4.9). Fr.4.2 (34 mg) was separated on a column of silica gel (petroleum ether-acetone, 100:1) to obtain **8** (2 mg). Fr.4.4 (37 mg) was chromatographed on Sephadex LH-20 (60 × 2 cm, CHCl_3_-MeOH) and then purified using a silica gel column (petroleum ether–EtOAc, 20:1, 10:1, 9:1, 8:2) to afford **9** (3 mg). Fr.4.5 (431 mg) was subjected to a silica gel column (petroleum ether–EtOAc, 200:1, 100:1, 50:1, 20:1, 10:1, 9:1, 8:2) and further purified by Sephadex LH-20 (60 × 2 cm, acetone) to obtain **11** (47 mg). Fr.4.6 (129 mg) was subjected on Sephadex LH-20 (60 × 2 cm, MeOH) and purified by a silica gel column (petroleum ether–EtOAc, 200:1, 100:1, 50:1, 20:1, 10:1, 9:1, 8:2) to obtain **3** (4 mg). Fr.4.7 (98 mg) was separated on Sephadex LH-20 (60 × 2 cm, acetone) and purified by preparative thin-layer chromatography (petroleum ether–EtOAc, 10:1) to afford **12** (2 mg). Fr.4.8 (105 mg) was subjected on a silica gel column (60 × 2 cm; petroleum ether–EtOAc, 50:1, 20:1, 10:1, 9:1, 8:2) and then purified by a silica gel column (60 × 2 cm; petroleum ether-acetone, 50:1, 20:1, 10:1, 9:1, 8:2;) to give **1** (10 mg). Fr.7 (3.4 g) was separated on a silica gel column (100 × 6 cm; CHCl_3_-MeOH, 200:1, 100:1, 50:1, 20:1, 10:1, 9:1, 8:2, 7:3) to obtain six fractions (Fr.7.1–Fr.7.6). Fr.7.3 (29 mg) was subjected on a silica gel column (60 × 2 cm; CHCl_3_-MeOH, 10:1, 9:1, 8:2, 7:3, 6:4) and then purified by Sephadex LH-20 (60 × 2 cm, MeOH) to obtain **10** (5 mg). Fr.7.5 (103 mg) was separated on a silica gel column (60 × 2 cm; CHCl_3_-acetone containing 0.1% formic acid, 50:1, 20:1, 10:1, 9:1, 8:2) to obtain 2 fractions (Fr.7.5.1 and Fr.7.5.2). Fr.7.5.1 (35 mg) was purified by a silica gel column (60 × 2 cm; CHCl_3_-acetone, 20:1, 10:1, 9:1, 8:2, 7:3) to obtain **4** (12 mg). Fr.7.5.2 was separated on Sephadex LH-20 (60 × 2 cm, MeOH) to obtain **6** (4 mg). Fr.8 (8.0 g) was separated on a RP-18 silica gel column (200 × 6 cm; H_2_O-MeOH, 100:0, 10:1, 7:3, 1:1, 3:7, 1:9, 0:100) to obtain five fractions (Fr.8.1–Fr.8.5). Fr.8.2 (3.72 g) was separated on a silica gel column (100 × 4 cm; CHCl_3_-MeOH, 200:1, 100:1, 50:1, 20:1, 10:1, 9:1, 8:2, 7:3) to obtain four fractions (Fr.8.2.1- Fr.8.2.4). Fr.8.2.3 (1.5 g) was subjected on a silica gel column (100 × 4 cm; CHCl_3_-acetone, 100:1, 50:1, 20:1, 10:1, 9:1, 8:2, 7:3) and purified by a silica gel column (60 × 2 cm; petroleum ether-acetone, 50:1, 20:1, 10:1, 9:1, 8:2, 7:3) to obtain **5** (3 mg). Fr.8.3 (500 mg) was separated on Sephadex LH-20 (100 × 2 cm, CHCl_3_-MeOH), chromatographed on a silica gel column (100 × 4 cm; CHCl_3_-MeOH, 200:1, 100:1, 50:1, 20:1, 10:1), and then purified by Sephadex LH-20 (60 × 2 cm, acetone) to obtain **7** (3 mg). Fr.8.6 (374 mg) was subjected on Sephadex LH-20 (100 × 2 cm, CHCl_3_-MeOH), separated on a silica gel column (60 × 2 cm; CHCl_3_-MeOH, 50:1, 20:1, 10:1, 9:1, 8:2, 7:3), and then purified using a Sephadex LH-20 column (60 × 2 cm, acetone) to obtain **2** (6 mg).

### 2.7. Spectroscopic Data for New Compounds

10-Hydroxyoctadecanoic acid (**1**): colorless amorphous; ${\left[\alpha \right]}_{D}^{23}$ = 2.8 (*c* = 0.10, MeOH); UV (MeOH) λ_max_ (log ε) nm: 203 (3.06), 273 (2.16); NMR data (see Table 1); ESI-MS: 323 [M + Na]^+^, 299 [M − H]^−^; HR-ESI-MS: 299.2581 ([M − H]^−^, calc. 299.2581).

9,10-Dihydroxyoctadecanoic acid (**2**): colorless amorphous; ${\left[\alpha \right]}_{D}^{23}$ = 4.6 (*c* = 0.10, MeOH); UV (MeOH) λ_max_ (log ε) nm: 203 (2.92), 281 (2.00); NMR data (see Table 1); ESI-MS: 339 [M + Na]^+^, 315 [M − H]^−^; HR-ESI-MS: 315.2536 ([M + Na]^+^, calc. 315.2530).

(*Z*)-10-hydroxyoctadec-12-enoic acid (**3**): colorless amorphous; ${\left[\alpha \right]}_{D}^{23}$ = −2.80 (*c* = 0.10, MeOH); UV (MeOH) λ_max_ (log ε) nm: 202 (3.00), 277 (1.90); NMR data (see Table 1); ESI-MS: 321 [M + Na]^+^, 297 [M − H]^−^; HR-ESI-MS: 297.2427 ([M − H]^−^, calc. 297.2424).

(22*E*,24*R*)-3β,5α,9α-trihydroxyergosta-7,22-dien-6-one-3-yl formate (**4**): colorless amorphous; ${\left[\alpha \right]}_{D}^{19}$ = −13.3 (*c* = 0.10, MeOH); UV (MeOH) λ_max_ (log ε) nm: 200 (3.72), 240 (3.91), 333 (2.10); NMR data (see Table 2); ESI-MS: 495 [M + Na]^+^; HR-ESI-MS: 495.3080 ([M + Na]^+^, calc. 495.3081).

5α,6α-Epoxy-(22*E*,24*R*)-ergosta-8,22-diene-3β,7α-diol-3-yl formate (**5**): colorless amorphous; ${\left[\alpha \right]}_{D}^{19}$ = 12.7 (*c* = 0.10, MeOH); UV (MeOH) λ_max_ (log ε) nm: 202 (3.53), 241 (3.21); NMR data (see Table 2); ESI-MS: 479 [M + Na]^+^; HR-ESI-MS: 479.3128 ([M + Na]^+^, calc. 4479.3132).

### 2.8. Assay of Nematicidal Activity against M. incognita

The twelve metabolites isolated from the *P. lavendulum* YMF1.00683 were used to test for nematicidal activity [19], with stearic acid and linoleic acid purchased from Macklin used as references. The metabolites were dispersed in MeOH, while stearic acid (SA) and oleic acid (OA) were dispersed in acetone. Two hundred J2s (10 μL) of *M*. *incognita* were added to each sample, and the concentration of the working solutions of the tested compounds was set to 400 ppm. The total and dead nematode numbers were determined every 24 h. Three replicates were conducted for each test.

### 2.9. Assay of Chemotaxis Activity against M. incognita

The nematode chemotaxis assay was conducted for the 12 isolated metabolites using the plate method [20]. The pure compounds were dispersed in MeOH to afford stock solutions with concentrations of 400 μg mL^−1^. These stock solutions were used to prepare working solutions (40, 20, and 10 ppm). After air drying, the sample was treated with 5 μL of the working solution, and MeOH (5 μL) was used as the control. Typically, 10 μL (~200 nematodes) of the solution was added to the middle of the plate. The chemotaxis index (CI) was calculated as reported elsewhere [20].

## 3. Results

### 3.1. Transcriptomics Analysis of C. elegans Infection by P. lavendulum

**Figure 1:**
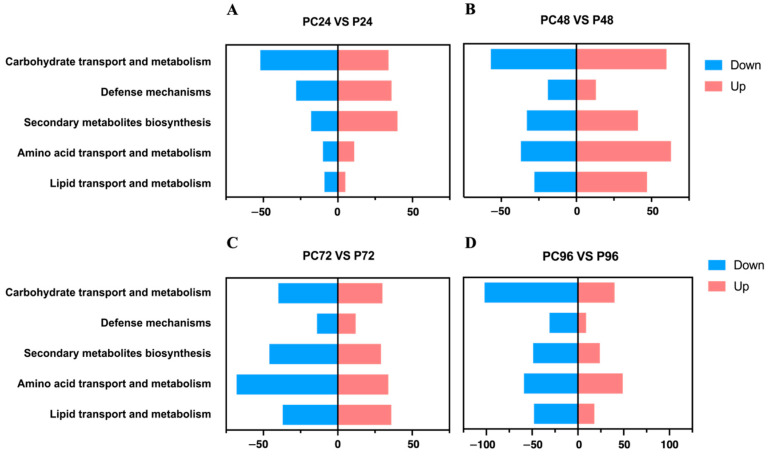
KEGG enrichment of differential genes for (**A**) 24 h, (**B**) 48 h, (**C**) 72 h, and (**D**) 96 h co-culturing.

To determine the changes induced by the interaction of *P.* *lavendulum* with *C*. *elegans*, we performed RNA sequencing experiments on isolated *P.* *lavendulum* and *P*. *lavendulum* interacting with *C*. *elegans*. As a result, 1160, 1533, 1012, and 1580 differentially expressed genes (DEGs) were identified in the latter (interaction) sample at 24, 48, 72, and 96 h (Figure 1). Among them, 434, 857, 482, and 706 genes were upregulated, respectively. The DEGs were divided into different categories. The GO classification of the DEGs showed that most of them were responsible for the basic processes related to biological regulation and metabolism, revealing significant differences in the transcription of genes involved in energy metabolism and transport (Figure 1). Notably, 45 of the DEGs were different at four stages from the beginning to the end of co-culturing, among which the genes encoding chitin synthase and phosphatidyltransferase showed differences in expression at each stage. In the early stage of infection, the expression of the chitin synthase and phosphatidyltransferase genes increased, as did the secretion of chitinase and phospholipase, which enhanced the degradation of the nematode epidermis. At the late stage of infection, epidermal degradation was complete, and the expression level decreased.

**Figure 2:**
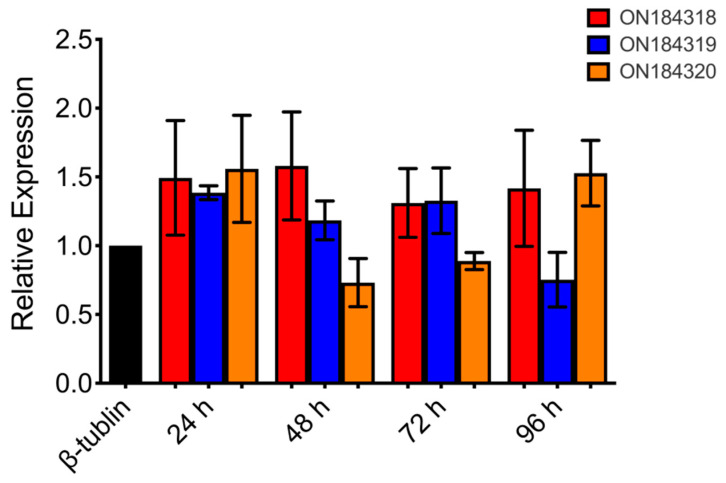
Relative transcription levels of three genes quantified using RT-qPCR at different interaction times. β-Tubulin was used as the standard for the statistical analysis of the transcription level of three genes in different co-culture times relative to those in the wild-type strain under a given condition. Error bars indicate the standard deviations.

Whereas carbohydrate decomposition-related genes were significantly downregulated during predation, ON184318, encoding a linoleate diol synthase, was significantly upregulated at 24 h. The linoleate diol synthase is involved in lipid metabolism, catalyzing the oxygenation of linoleate, and plays important roles in plant development and defense responses under various environmental stresses. Much evidence suggests that oxylipin is an important factor that regulates biological development, participates in cellular signaling pathways [21,22], and plays an important role in the defense response against pathogens [23,24,25]. The transcriptomic data analysis of *C. elegans* infection by *P. lavendulum* showed that the transcription of the gene encoding chitinase (ON184319) was upregulated, which agreed with the known upregulation of chitinases at the transcriptome level during the fungal infection of nematodes [26,27]. Another gene (ON184320) encoding 3-hydroxy-3-methylglutaryl-coenzyme A reductase, which is a rate-limiting enzyme in the mevalonate pathway, plays a key role in the biosynthesis of terpenoids and steroids in fungi. Therefore, the above three genes were selected to verify changes in their transcription level by RT-qPCR. The results showed that the mRNA levels of the linoleate diol synthase in the interaction sample increased to 149.3, 158.0, 131.1, and 141.7% at 24, 48, 72, and 96 h, respectively (Figure 2). The mRNA levels of chitinase in the interaction sample also slightly increased at 24, 48, and 72 h but decreased at 96 h (Figure 2).

### 3.2. UPLC-MS Analysis of Interaction Samples between P. lavendulum and C. elegans

**Figure 3:**
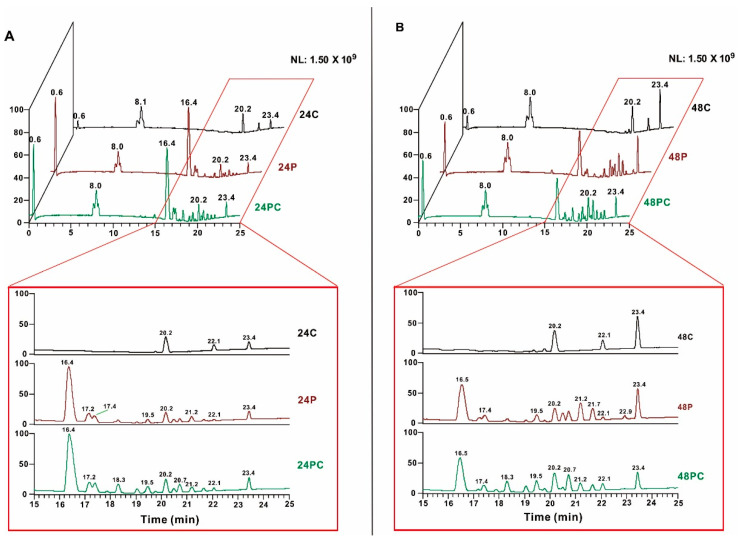
Chromatograms of interaction and control samples. (**A**) Base peak chromatograms of samples at 24 h: 24PC = experimental group, 24P and 24C = control groups. Red box shows chromatogram expansions. (**B**) Base peak chromatograms of samples at 48 h: 48PC = experimental group, 48P and 48C = control groups. Red box shows chromatogram expansions.

In order to investigate whether the metabolites of *P. lavendulum* in the process of infecting nematodes are the same as those under the same culture conditions, samples were collected at 24, 48, 72, and 96 h after infection (PC group) and analyzed by UPLC-MS, with the group P and group C samples used as controls. To better visualize the differences in the changes of the main components, we used base peak chromatograms. As a result, several peaks appeared (17.9 and 19.8 min) or gained intensity (17.9, 18.3, 19.1, 19.8, and 20.7 min) at retention times of 15–24 min in the 24 h PC group (Figure 3A). Similar results were observed for the 48 h PC samples (Figure 3B). At 72 and 96 h, changes in the main components were still present, but the metabolites of the PC group were not significantly different from those of the control samples. Thus, from the perspective of the main components, the process of nematode infection by *P. lavendulum* promotes the biosynthesis of some compounds. The UPLC-MS data of the PC group revealed several peaks with enhanced abundances, namely, those with *m*/*z* 299.2576, 321.2399, 339.2494, 339.2887, and 467.3126 and retention times of 17–24 min.

As the transcriptomic and RT-qPCR analyses showed that the transcription of the lipoxygenase gene was significantly upregulated at 24 h, we analyzed the possible fatty acid metabolites. Compared with the control (C and P) groups, the PC group showed different quasi-molecular ion peaks (*m*/*z* 299.2576 and 339.2494), which were ascribed to the products of fatty acid oxidation. After repeated experiments, some specific small molecules were stably identified. Although the relative abundances of some metabolites significantly increased during the infection process, these metabolites could not be obtained in large amounts at this stage. The results of the UPLC-MS analysis showed that, although some metabolites in the PC group were obviously produced, the metabolites also accumulated and changed with time in the P group. Therefore, in the next step, we screened culturing conditions to identify those favoring the production of metabolites corresponding to oxidized fatty acids.

### 3.3. Results of Culturing Conditions Screening

**Figure 4:**
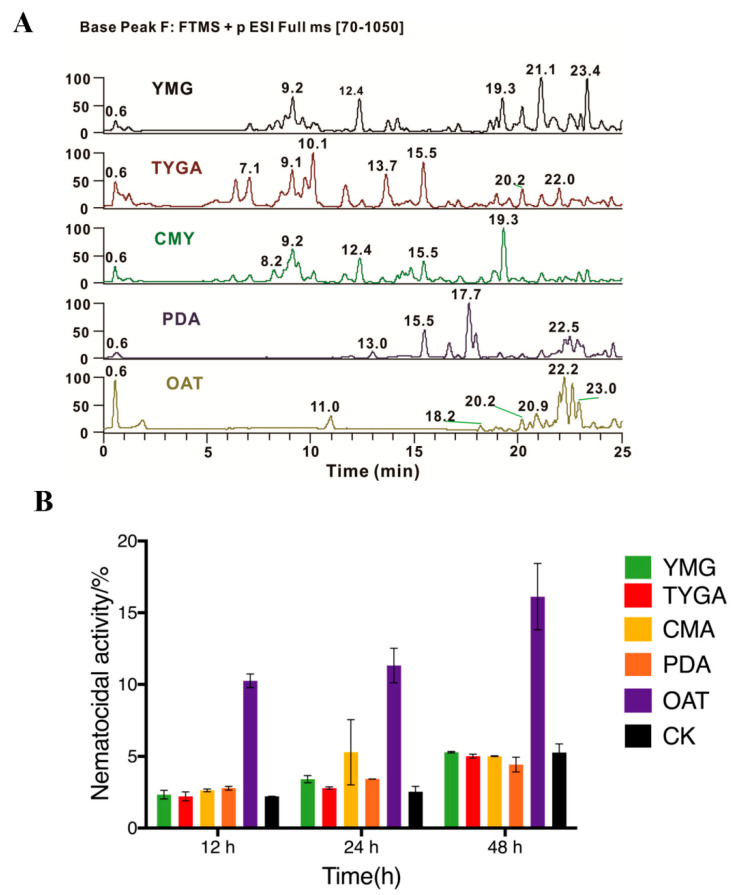
Results of culture medium screening based on UPLC profiling and nematicidal activity assessment. (**A**) Profiles of crude extracts produced through the fermentation of *P*. *lavendulum* on five solid media. (**B**) Nematicidal activities of crude extracts obtained from different media.

As the interaction experiment was carried out using a solid medium, we selected five solid media to ferment *P. lavendulum* for screening culturing conditions, producing compounds similar to those in the PC group. After the 21-day culturing of *P. lavendulum* on solid media, the extracts were analyzed by LC-MS and assayed to determine nematicidal activity. The results of the LC-MS analysis showed that the chromatogram profiles of the samples cultured on oat and PDA media resembled those of the PC group, featuring peaks corresponding to fewer polar compounds with retention times of 16–25 min. In the case of the oat solid medium, quasi-molecular ion peaks with *m*/*z* 299.2576, 321.2399, and 339.2494 were detected, gaining intensity in the PC group. Moreover, the metabolites produced on the former medium were more uniformly distributed on the chromatogram (Figure 4A). Finally, the nematicidal activity of the five extracts was highest in the case of the oat medium (Figure 4B). Therefore, we chose the oat solid medium to expand the mycelium of *P. lavendulum* and isolate and identify target compounds.

### 3.4. Identification of Compound Structures

Compound **1** was obtained as colorless and amorphous. The results of the negative HR-ESI-MS analysis indicated a molecular formula of C_18_H_36_O_3_ based on the [M − H]^−^ ion peak at *m*/*z* 299.2579 (calc. 299.2581).

**Figure 5:**
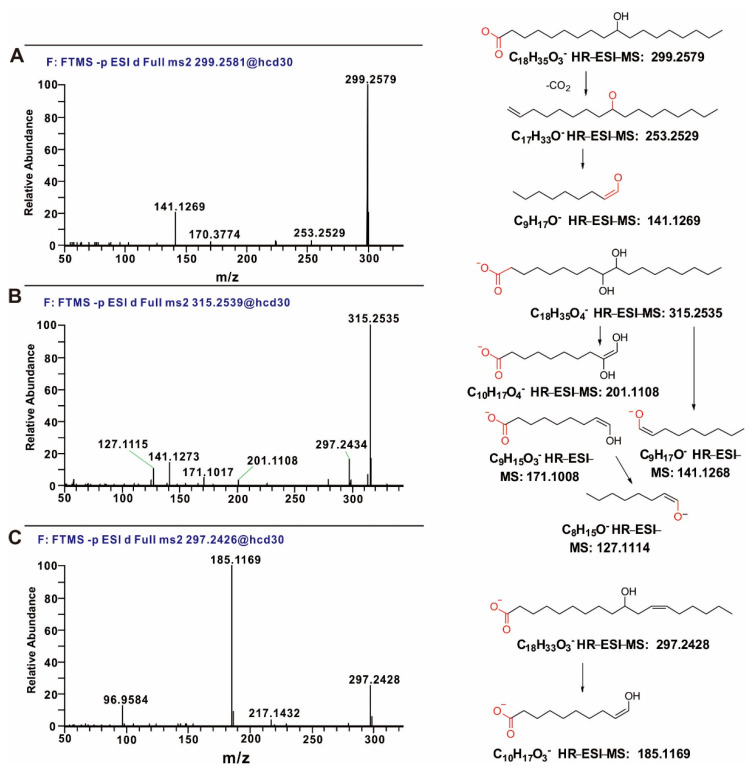
The MS/MS spectra of compounds **1–3.** (**A**). The HR-ESI-MS and MS/MS spectra and fragmentation pathway for compound **1**. The HR-MS/MS spectrum of **1** showed the parent ion with *m*/*z* 299.2579 and two product ions with *m*/*z* 253.2529 and 141.1269. And Proposed fragmentation pathways for compound **1** producing *m*/*z* 253.2529 and 141.1269. (**B**). The HR-ESI-MS and MS/MS spectra and fragmentation pathway for compound **2**. The HR-MS/MS spectrum of **2** showed the parent ion with *m*/*z* 315.2535 and several key product ions with *m*/*z* 201.1108, 141.1273 and 127.1115. And Proposed fragmentation pathways for compound **2** producing *m*/*z* 201.1108, 141.1273 and 127.1115. (**C**). The HR-ESI-MS and MS/MS spectra and fragmentation pathway for compound **3**. The HR-MS/MS spectrum of **3** showed the parent ion with *m*/*z* 297.2428 and the key product ion with *m*/*z* 185.1169. And Proposed fragmentation pathways for compound **3** producing *m*/*z* 185.1169.

**Figure 6:**
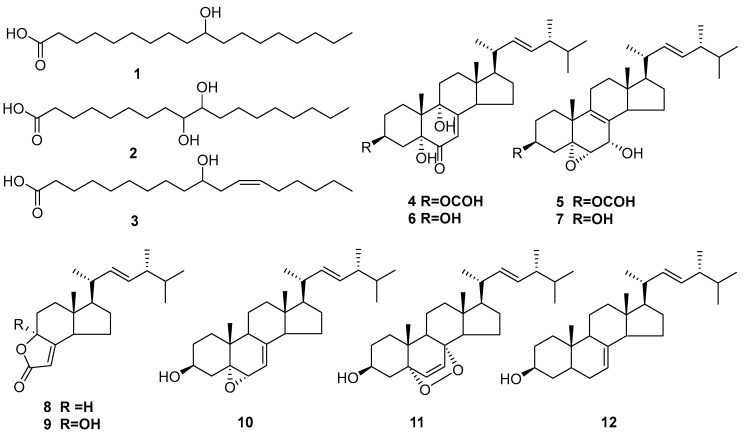
The structures were identified from *P. lavendulum*.

The ^13^C-NMR and DEPT spectra (Table 1) revealed one quaternary carbon (*δ*_C_ 178.8), one oxygen-substituted methine, fifteen methylene groups, and one methyl group (*δ*_C_ 14.1). According to MS and NMR data, compound **1** was presumed to be an 18-carbon straight-chain fatty acid bearing one hydroxyl group. Despite the availability of 2D-NMR data, we could not identify the position of the hydroxyl group because of the overlap of numerous NMR signals and therefore resorted to HR-ESI-MS and MS/MS data to obtain structural clues. The MS/MS spectrum of **1** (Figure 5A) featured the parent ion and two product ions with *m*/*z* 253.2529 and 141.1269. The ion with *m*/*z* 253.2529 was assumed to have the composition of C_17_H_33_O^−^ ([M − H]^−^ calc. for 253.2526) and was produced through the sequential loss of CO_2_ and H_2_ from the parent ion (*m*/*z* 299.2579), which afforded a stable alkoxide anion. This fragmentation is believed to be typical of hydroxylated fatty acids [28]. The ion with *m*/*z* 141.1269 was assumed to have the composition of C_9_H_17_O^−^ (calc. for 141.1274) and could be produced via (i) homolytic C9–C10 cleavage with subsequent C11–H bond homolysis or (ii) the loss of CO_2_ from the parent ion (*m*/*z* 299.2581) followed by C10–C11 bond homolysis and the loss of a C9 hydrogen radical to form *m*/*z* 141.1274 [28,29]. Thus, the hydroxyl group was located at C10, i.e., **1** was identified as 10-hydroxyoctadecanoic acid (Figure 6).

Compound **2** was obtained as a colorless, amorphous solid. Negative HR-ESI-MS data indicated a molecular formula of C_18_H_35_O_4_ based on the [M − H]^−^ ion with *m*/*z* 315.2536 (calc. 315.2530). According to MS and NMR data, **2** was identified as doubly hydroxylated octadecanoic acid. The exact positions of the hydroxyl groups were elucidated using MS/MS. The MS/MS spectrum of **2** (Figure 5B) featured the peak of the parent ion as well as those of five product ions with *m*/*z* 297.2434, 201.1108, 171.1017, 141.1273, and 127.1115. Among them, the ion of *m*/*z* 297.2334 was produced through the loss of one hydroxyl group not involving C–C bond cleavage, whereas the other four ions could be formed through C–C bond cleavage and provide clues for determining the hydroxyl group positions. According to the literature, the fragmentation of fatty acids with multiple hydroxyl groups involves the cleavage of bonds between the hydroxyl-substituted C and its neighboring (α) atoms [28,30,31]. The key product ion with *m*/*z* 201.1108, identified as C_10_H_17_O_4_^−^ (calc. for 201.1121), could be produced through C10–C11 bond homolysis followed by C10–H homolytic cleavage, while the ion with *m*/*z* 171.1008 (C_9_H_15_O_3_^−^, calc. for 171.1016) was possibly produced through C9–C10 bond cleavage. Moreover, the ion with *m*/*z* 127.1114 (C_8_H_15_O^−^, calc. for 127.1117) was produced by the loss of CO_2_ from the parent ion (*m*/*z* 315.2535) followed by C9–C10 bond homolysis and the subsequent loss of a C9 hydrogen radical. According to the above MS/MS data, **2** was identified as 9,10-dihydroxyoctadecanoic acid (Figure 6).

Compound **3** was obtained as a colorless, amorphous solid. Negative HR-ESI-MS data suggested a molecular formula of C_18_H_33_O_3_ based on the [M − H]^−^ ion with *m*/*z* 297.2427 (calc. 297.2424). According to MS and NMR data, **3** was identified as octadecanoic acid containing one hydroxyl group and one double bond. Further structural insights were provided by HSQC and HMBC experiments. In particular, the HMBC experiment showed that the methylene protons at *δ*_H_ 2.19 were correlated with the carbons at *δ*_C_ 36.8, 71.5, 125.0, and 133.6, while the methylene protons at *δ*_H_ 2.04 were correlated with carbons at *δ*_C_ 29.0, 125.0, and 133.6. These data revealed that the hydroxylated carbon and the double bond were connected in *meta* positions and were separated by a methylene group (*δ_H_* 2.19, *δ_C_* 35.3). In the MS/MS spectra of fatty acids with double bonds and hydroxyl groups, the main product ions are generally generated via cleavage adjacent to the carbinol C–C bond at the allylic position [32,33,34]. The MS/MS spectrum of **3** (Figure 5C) featured a distinct product ion with *m*/*z* 185.1169, which was assumed to have the composition of C_10_H_17_O_3_^−^ (calc. 185.1172) and could be produced via C10–C11 bond homolysis with the subsequent homolysis of the C10–H bond. Thus, based on 2D-NMR and MS/MS data, **3** was identified as (*Z*)-10-hydroxyoctadec-12-enoic acid (Figure 6).

Compound **4** was obtained as a colorless, amorphous solid. Positive HR-ESI-MS data suggested a molecular formula of C_29_H_44_O_5_Na based on the [M + Na]^+^ ion with *m*/*z* 495.3080 (calc. 495.3081). ^13^C-NMR and DEPT spectra (Table 2) revealed seven quaternary carbons (*δ*_C_ 197.4_,_ 164.9, 160.9, 79.4, 74.5, 45.3, and 41.8), nine methine groups (*δ*_C_ 135.0, 132.4, 119.5, 70.5, 55.9, 51.8, 42.7, 40.3, and 33.1), seven methylene groups (*δ*_C_ 34.9, 33.4, 28.7, 27.9, 26.2, 25.2, and 22.4), and six methyl groups (*δ*_C_ 21.1, 20.2, 19.9, 19.6, 17.6, and 12.3). The combined ^1^H-, ^13^C- and DEPT NMR data (Table 2) indicated that **4** was a sterol. Compound **4** was very similar to **6**, except for an additional group appended to the 3-OH of the latter compound (Figure 6), which resulted in a downfield shift of the corresponding signals (from *δ_H_* 4.62 and *δ_C_* 66.8 in **6** to *δ_H_* 5.25 and *δ_C_* 70.5 in **4**) [35]. HMBC data showed that the proton at *δ*_H_ 8.02 (3-OCHO) was correlated with the carbon at *δ*_C_ 70.5 (C-3). Based on the similarity of the NMR data and the biogenetic perspective [35], **4** was proposed to have the absolute configuration shown in Figure 6 ((22*E*,24*R*)- 3β,5α,9α-trihydroxyergosta-7,22-dien-6-one-3-yl formate).

Compound **5** was obtained as a colorless, amorphous solid. Positive HR-ESI-MS data indicated a molecular formula of C_29_H_44_O_4_Na based on the [M + Na]^+^ ion with *m*/*z* 479.3128 (calc. 479.3132). The acquired NMR data suggested that **5** was a steroid featuring a formate group attached to the 3-hydroxyl group (Table 2). HMBC data showed that the proton at *δ*_H_ 8.01 (3-OCHO) was correlated with the carbon at *δ*_C_ 71.0 (C-3). Based on the similarity of the NMR data and the biogenetic perspective [36], **5** was proposed to have the absolute configuration shown in Figure 6 (5α,6α-epoxy-(22*E*,24*R*)-ergosta- 8,22-diene-3β,7α-diol-3-yl formate).

Other compounds were determined to be (22*E*,24*R*)-3β,5α,9α-trihydroxy-ergosta- 7,22-dien-6-one (**6**) [35], 5α,6α-epoxy-(22*E*, 24*R*)-ergosta-8,22-diene-3β,7α-diol (**7**) [36], (3a*S*,5a*R*,6*R*,8a*R*)-3a,4,5,5a,6,7,8,8a-octahydro-5a-methyl-6-[(1*R*, 2*E*,4*R*)-1,4,5-trimethyl-2- hexen-1-yl]-2H-indeno[5,4-b]furan-2-one (**8**) [37], demethylincisterol A_3_ (**9**) [37], 5α,6α-epoxy-24(*R*)-methylcholesta-7,22-dien-3β-ol (**10**) [38], 5α,8α-epidioxy- (22*E*)-ergosta- 6,22-dien-3β–ol (**11**) [39], and stella sterol (**12**) [40].

### 3.5. Effect of Metabolites on the Mortality of M. incognita J2s

**Figure 7:**
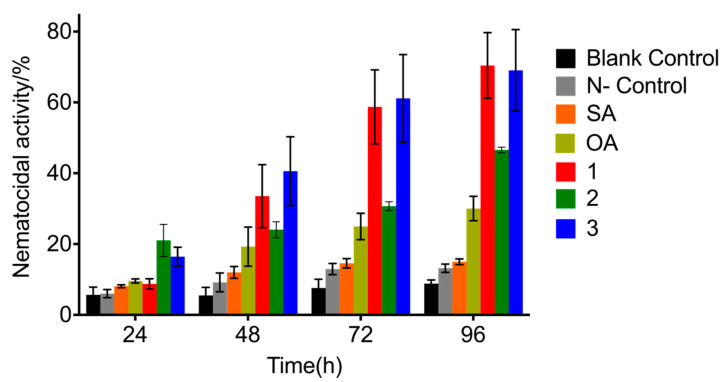
Nematicidal activities of metabolites isolated from *P*. *lavendulum*. Mortality (%) of *M*. *incognita* J2s treated with OA, SA, and **1**–**3**.

Compounds **1**–**3** isolated from *P. lavendulum* YMF1.00683 had different nematicidal activities against the J2s of *M.* *incognita* (Figure 7). At a test concentration of 400 ppm, **2** and **3** showed weak nematicidal activity at 24 h, while compound **1**, SA, and OA had no obvious nematicidal activity. At 48, 72, and 96 h, the nematicidal activities of **1** and **3** exceeded those of SA and OA. At 96 h, mortalities of 70.4, 48.6, 69.1, 30.0, and 15.0% were observed for **1**, **2**, **3**, OA, and SA, respectively. Fatty acids, which are ubiquitous in nature and play a key role in life processes, show antifungal activities, inhibit pathogenic bacteria, and activate plant disease resistance. In addition, fatty acids (e.g., caproic, caprylic, capric, lauric, myristic, and palmitic acids) are known to be toxic to the J2s of *M. incognita* [19]. Moreover, a mixture of fatty acids containing linoleic acid, OA, and palmitic acid isolated from *Pleurotus pulmonarius* was reported to exhibit nematicidal activity [41].

### 3.6. Chemotactic Activities of Metabolites

**Figure 8:**
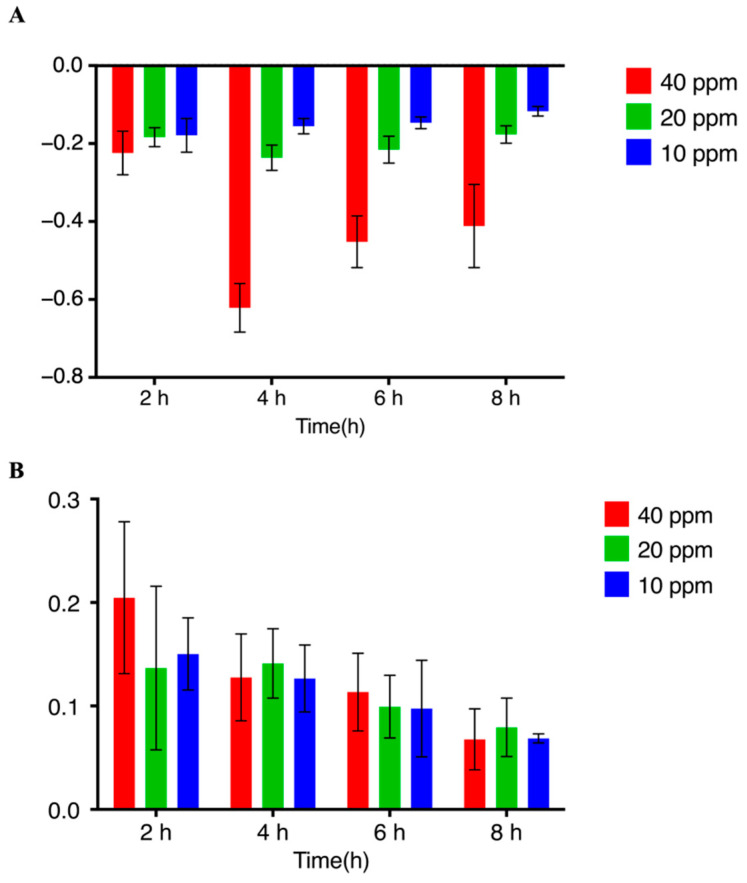
Results of the nematode chemotaxis assay. (**A**) Avoidance activity of **8** at different times and concentrations. (**B**) Attractive activity of **9** at different times and concentrations.

The *M. incognita* J2s exhibited negative chemotaxis toward **8**, whereas the opposite effect was observed for **9**, and the other tested metabolites featured no pronounced activity. At a concentration of 40 ppm, **8** exhibited a CI of −0.65 at 4 h, losing activity at 8 h; meanwhile, the effect of **8** became less pronounced as its concentration decreased from 40 to 10 ppm (Figure 8A). At 40 ppm and 2 h, **9** showed a CI of 0.21, and the activity of the compound decreased with decreasing time and concentration (Figure 8B).

## 4. Discussion

The microbial control of nematodes is a sustainable and environmentally friendly strategy. However, the effects and persistence of some biocontrol microorganisms with potential application value vary greatly with region, soil characteristics, and agricultural practice. However, a more important reason may be that the molecular mechanism of the microbial infection of nematodes is not fully understood [42]. Currently, it is accepted that nematophagous fungi need to break through the cuticle or eggshell of plant-parasitic nematodes during infestation to digest the internal tissues of their hosts. The invasion mechanism may be completed by mechanical pressure and enzymatic hydrolysis. Proteases such as chitinase, protease, and lipase, which are often the determinants of host-infected pathogenic strains, may play key roles in fungal infection. In the transcriptome of the infection of *C. elegans* by *P. lavendulum*, the transcription of genes corresponding to chitinase, protease, and esterase was significantly upregulated.

In addition, some microorganisms with biocontrol potential secrete small molecular compounds to attract nematodes and further kill them. For example, *Bacillus nematocida* B16, which has good application prospects, produces 2-heptanone to attract nematodes, subsequently infesting and killing them through protease secretion [43,44], while *Arthrobotrys oligospora* produces volatile methyl 3-methyl-2-butenoate to attract *C. elegans* and realizes a nematode trapping strategy [45]. Two Zn(2)-C6 transcription factors responsible for the regulation of fungal metabolism were significantly upregulated during the interaction between *P. lavendulum* and *C. elegans*. The metabolite difference analysis of the interaction between fungi and nematodes showed that some small molecular metabolites were involved. Based on the transcriptome data and metabolite differences corresponding to the interaction between *P. lavendulum* and nematodes, 12 compounds were obtained using an optimized oat solid medium. The experiments evaluating the effect of the secondary metabolites of *P. lavendulum* on the chemotaxis of *M. incognita* revealed that **9** had a moderate attractive activity at 40, 20, and 10 ppm, with the respective CI values equaling 0.21, 0.15, and 0.16. Compound **8** showed avoidance activity at 40, 20, and 10 ppm, with the respective CI values equaling −0.64, −0.23, and −0.18. Although **8** and **9** are both steroid degradation products [37,46] with structural differences limited to substituents at the 4-position, these differences are sufficient to afford opposite activities.

In the nematicidal activity assay, **1** and **3** showed toxicity against *M. incognita* (70.4 and 69.1% mortalities, respectively) at 400 ppm. These compounds are the precursors of oxylipins, which are a large class of oxidized fatty acids and their derived metabolites widely found in animals, plants, bacteria, and fungi. In plants, oxylipins act as signaling molecules to regulate developmental processes such as pollen formation and mediate responses to biotic and abiotic stresses such as herbivore or pathogen attack and desiccation [47,48]. Oxylipins are enzymatically formed by the initial peroxidation of polyunsaturated fatty acids catalyzed by lipoxygenase. However, some studies report the direct production of jasmonic acid and its derivatives by fungi such as the phytopathogenic fungus *Botrydiplodia theobromae* [49], while this acid and its derivatives were also found in *Fusarium oxysporum* [50]. Although numerous hydroxylated fatty acids and their derivatives have been isolated from microorganisms, their functions remain unclear [51]. During the interaction between *P. lavendulum* and *C. elegans*, lipoxygenase was upregulated to varying degrees at 48 and 72 h, which indicated that, when infecting nematodes, the fungus could also produce hydroxylated fatty acids through the oxygen–lipid pathway. Thus, mycotoxins or other secondary metabolites harmful to nematodes may be a selective strategy of fungi against prey.

## Figures and Tables

**Table 1:** The NMR data of compounds **1**–**3**.

Position	1		2		3	
	^1^H	^13^C	^1^H	^13^C	^1^H	^13^C
1	-	178.8	-	177.8	-	178.9
2	2.36 (2H, t, *J* = 7.5 Hz)	33.9	2.26 (2H, t, *J* = 7.4 Hz)	35.0	2.32 (2H, t, *J* = 7.4 Hz)	33.9
3	1.64 (2H, t, *J* = 7.1 Hz)	24.7	1.60 (2H, m)	26.1	1.62 (2H, m)	24.6
4	1.21–1.40 (2H, m)	29.7	1.30–1.50 (2H, m)	30.5	1.18–1.35 (2H, m)	29.7
5	1.21–1.40 (2H, m)	29.0	1.30–1.50 (2H, m)	30.74	1.18–1.35 (2H, m)	29.5
6	1.21–1.40 (2H, m)	29.1	1.30–1.50 (2H, m)	30.9	1.18–1.35 (2H, m)	25.5
7	1.42 (2H, m)	25.63	1.30–1.50 (2H, m)	27.1	1.42–1.46 (2H, m)	36.8
8	1.42 (2H, m)	37.4	1.30–1.50 (2H, m)	33.88	3.59 (1H, m)	71.5
9	3.60 (1H, m)	72.1	3.37 (1H, brd, *J* = 8.5 Hz)	75.31	2.19 (2H, t, *J* = 7.4 Hz)	35.3
10	1.42 (2H, m)	37.5	3.37 (1H, brd, *J* = 8.5 Hz)	75.28	5.59 (1H, dt, *J* = 10.8, 7.6 Hz)	125.0
11	1.21–1.40 (2H, m)	25.55	1.30–1.50 (2H, m)	33.90	5.59 (1H, dt, *J* = 10.8, 7.3 Hz)	133.6
12	1.21–1.40 (2H, m)	29.27	1.30–1.50 (2H, m)	27.0	2.04 (1H, dt, *J* = 10.8, 7.3 Hz)	27.4
13	1.21–1.40 (2H, m)	29.32	1.30–1.50 (2H, m)	30.2	1.18–1.35 (2H, m)	29.0
14	1.21–1.40 (2H, m)	29.56	1.30–1.50 (2H, m)	30.67	1.18–1.35 (2H, m)	29.1
15	1.21–1.40 (2H, m)	29.58	1.30–1.50 (2H, m)	30.4	1.18–1.35 (2H, m)	29.2
16	1.21–1.40 (2H, m)	31.9	1.30–1.50 (2H, m)	33.1	1.18–1.35 (2H, m)	31.5
17	1.21–1.40 (2H, m)	22.7	1.30–1.50 (2H, m)	23.7	1.18–1.35 (2H, m)	22.5,
18	0.89 (3H, t, *J* = 6.9 Hz)	14.1	0.90 (3H, t, *J* = 6.8 Hz)	14.4	0.86 (3H, t, *J* = 6.7 Hz)	14.0

**Table 2:** The NMR data of compounds **4** and **5**.

Position	4		5	
	^1^H	^13^C	^1^H	^13^C
1	1.55 (1H, m)	25.2, t	1.25 (1H, m)	29.7, t
	2.46 (1H, dt, *J* = 3.9, 13.9 Hz)		1.70 (1H, m)	
2	1.25 (1H, m)	27.9, t	1.80 (2H, m)	29.0, t
	1.85 (1H, m)			
3	5.25 (1H, m)	70.5, d	5.10 (1H, m)	71.0, d
4	1.51 (1H, m)	33.4, t	1.55 (1H, m)	35.6, t
	2.16 (1H, d, *J* = 3.12 Hz)		2.33 (1H, t, *J* = 12.5 Hz)	
5	-	79.4, s	-	65.1, s
6	-	197.4, s	3.29 (1H, brd, *J* = 2.6 Hz)	63.3, d
7	5.65 (1H, s)	119.5, d	4.20 (1H, d, *J* = 6.4 Hz)	67.0, d
8	-	164.9, s	-	127.0, s
9	-	74.5, s	-	134.1, s
10	-	41.8, s	-	37.9, s
11	1.37 (1H, m)	28.7, t	2.05 (2H, m)	23.7, t
	1.76 (1H, m)			
12	1.73 (1H, m)	34.9, t	1.41 (1H, m)	35.6, t
	1.86 (1H, m)		1.96 (1H, m)	
13	-	45.3, s	-	42.0, s
14	2.77 (1H, dd, *J* = 6.9, 11.9 Hz)	51.8, d	2.18 (1H, m)	49.5, d
15	1.47 (1H, m)	22.4, t	1.27 (2H, m, overlap)	23.4, t
	1.60 (1H, m)			
16	1.37 (1H, m)	26.2, t	1.67 (1H, m)	27.0, t
	1.75 (1H, m)		2.11 (1H, m)	
17	1.43 (1H, m)	55.9, d	1.20 (1H, d, *J* = 9.4 Hz)	53.6, d
18	0.61 (3H, s)	12.3, q	1.15 (3H, s)	22.6, q
19	1.02 (3H, s)	20.2, q	0.58 (3H, s)	11.3, q
20	2.04 (2H, m)	40.3, d	2.05 (1H, m)	40.4, d
21	1.03 (3H, d, *J* = 6.6 Hz)	21.1, q	1.01 (3H, d, *J* = 6.4 Hz)	20.9, q
22	5.18 (1H, dd, *J* = 7.8, 15.3 Hz)	135.0, d	5.13 (1H, dd, *J* = 7.8, 15.6 Hz)	135.5, d
23	5.23 (1H, m)	132.4, d	5.23 (1H, dd, *J* = 7.6, 15.6 Hz)	132.0, d
24	1.91 (1H, m)	42.7, d	1.85 (1H, m)	42.8, d
25	1.45 (1H, m)	33.1, d	1.44 (1H, m)	33.0, d
26	0.92 (3H, d, *J* = 6.8 Hz)	17.6, q	0.92 (3H, d, *J* = 6.9 Hz)	17.6,q
27	0.83 (3H, d, *J* =6.7 Hz)	19.9, q	0.82 (3H, d, *J* = 6.9 Hz)	19.9, q
28	0.84 (3H, d, *J* = 6.8 Hz)	19.6, q	0.83 (3H, d, *J* = 6.8 Hz)	19.6, q
3-OCOH	8.02 (1H, s)	160.9, d	8.01 (1H, s)	160.7, d

## Data Availability

Not applicable.
